# P-243. Association Between Integrase inhibitor Use with Insulin Resistance and Incident Diabetes Mellitus in Persons Living with HIV: A Systematic Review and Meta-analysis

**DOI:** 10.1093/ofid/ofaf695.465

**Published:** 2026-01-11

**Authors:** Frank Mulindwa, Barbara Castelnuovo, Jean-Marc Schwarz, Robert C Bollinger, Nele Brusselaers

**Affiliations:** United Health Services, Wilson Hospital, Johnson City, NY; Makerere University Infectious Diseases Institute, Kampala, Kampala, Uganda; University of California San Francisco, San Francisco, California; Johns Hopkins, MD; University of Antwerp, Antwerp, Antwerpen, Belgium

## Abstract

**Background:**

Integrase based anti-retroviral therapy is currently the most used first- and second-line therapy for the treatment of HIV. Case reports and series of patients developing accelerated hyperglycaemia after initiating integrase inhibitors have been reported. Whether integrase inhibitors (INSTIs) are associated with a higher risk of incident type 2 diabetes mellitus than other antiretroviral therapies (ART) needs to be established.

Risk of incident diabetes mellitus and hyperglycemia with exposure to integrase inhibitors

**Figure:**
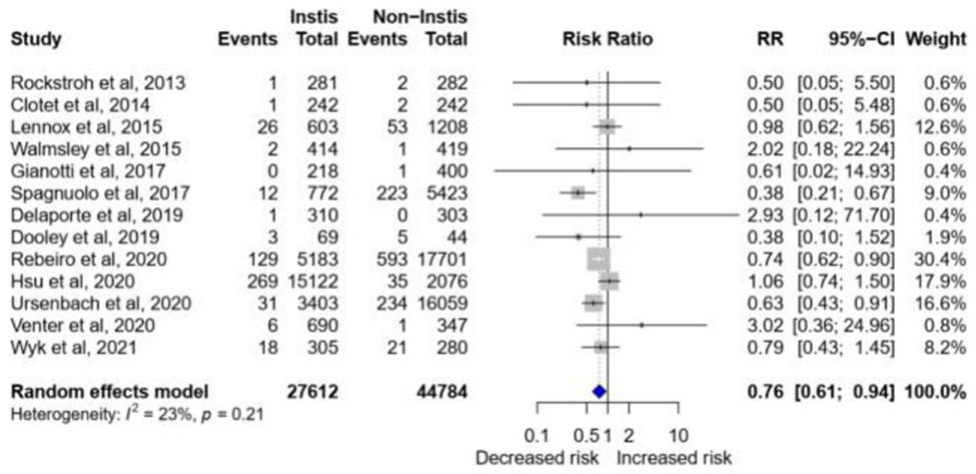

Sub-analysis for the risk of incident diabetes mellitus and hyperglycemia with exposure to integrase inhibitors

**Figure:**
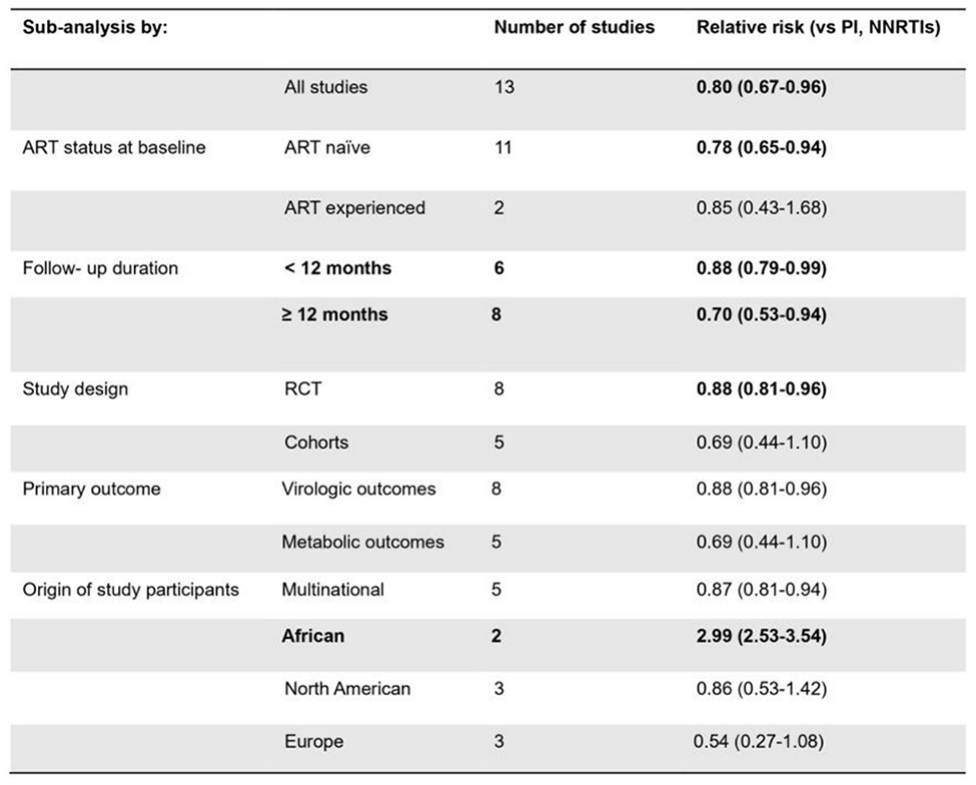

**Methods:**

Medline, Embase, Web of Science and ClinicalTrials.gov registries were searched for studies published between 1st January 2000 to 15^th^ June 2022. Eligible studies reported incident diabetes mellitus or mean changes in insulin resistance measured by homeostatic model index (HOMA-IR) in patients on INSTIs compared to other ARTs. We performed random-effects meta-analyses to obtain pooled relative risks (RR) with 95% confidence intervals (CI).

Mean changes in HOMA-IR from baseline in INSTIs group compared to overall non-INSTIs, NNRTIs and PIs groups.

**Figure:**
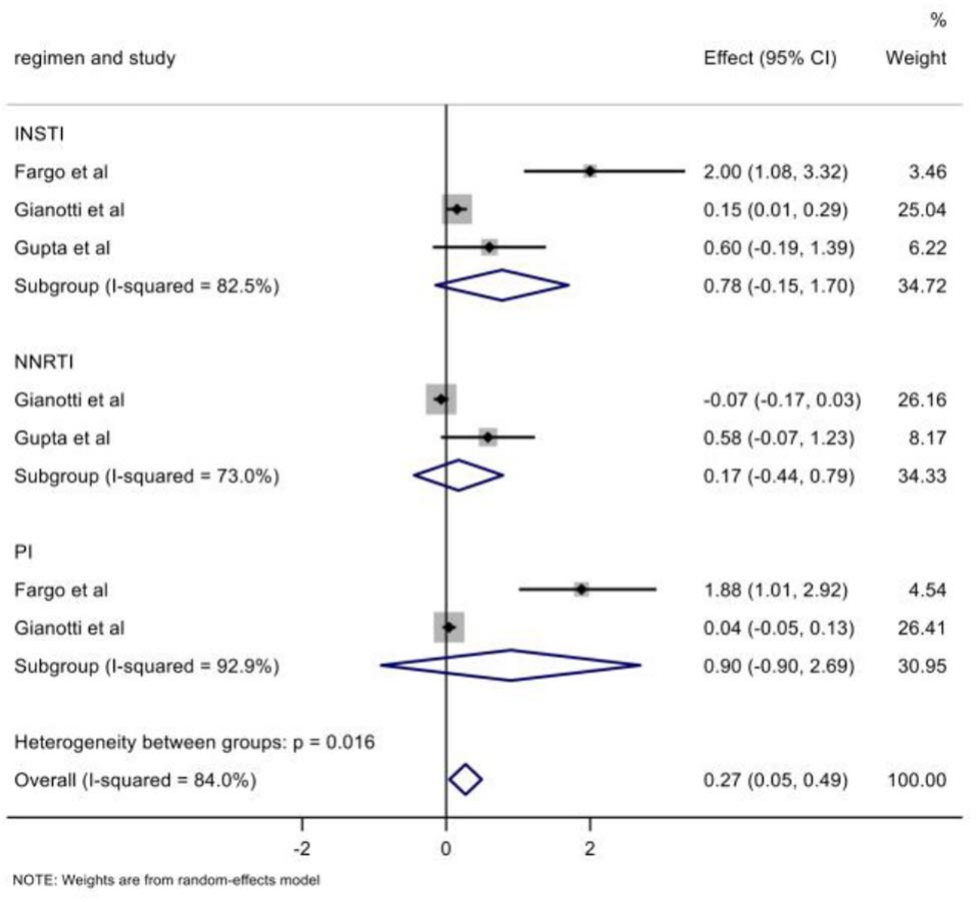

**Results:**

A total of 16 studies were pooled, 13 studies meta-analysed for incident diabetes with a patient population of 72,404 and 3 for changes in HOMA-IR. INSTI therapy was associated with a lower risk of incident diabetes in 13 studies (relative risk [RR] 0.80, 95% CI 0.67-0.96, I^2^=29%), of which 8 randomised controlled trials demonstrated a 22% reduced risk (RR 0.88, 95% CI 0.81-0.96, I^2^=0%). INSTIs had a lower risk compared to non-nucleoside reverse transcriptase inhibitors (NNRTI) (RR 0.75, 95% CI 0.63-0·89, I^2^=0%), but similar to protease inhibitor (PI)-based therapy (RR 0.78, 95% CI 0.61-1.01, I^2^=27%). The risk was lower in studies with longer follow-up (RR 0.70, 95% CI 0.53-0.94, I^2^=24%) and among ART naïve patients (RR 0.78, 95% CI 0.65-0.94, I^2^=3%) but increased in African populations (RR: 2.99, 95% CI: 2.53-3.54, I^2^=0%). INSTIs were associated with an insignificant increase in mean HOMA-IR from baseline compared to non-INSTIs (0.78, 95%CI -0.15 – 1.70) and PIs (0.90, 95%CI -0.90 – 2.69) and to NNRTIs (0.17, 95%CI -0.44 – 0.79)

**Conclusion:**

Exposure to INSTIs was not associated with increased risk of diabetes mellitus, except in African populations which were largely under-represented in the meta-analysis. Stratified analyses suggested reduced risk among ART naïve patients and studies with longer follow-up.

**Disclosures:**

Robert C. Bollinger, Jr., MD, MPH, [SCENE] Health: Advisor/Consultant|[SCENE] Health: Board Member|[SCENE] Health: Stocks/Bonds (Private Company)|Merck: Advisor/Consultant|miDiagnostics: Co-inventor of IP owned by Johns Hopkins University|miDiagnostics: Eligible for equity and royalty payments received by Johns Hopkins University

